# A Three-Point Hyperbolic Combination Model for the Settlement Prediction of Subgrade Filled with Construction and Demolition Waste

**DOI:** 10.3390/ma13081959

**Published:** 2020-04-22

**Authors:** Haiying Wang, Hui She, Jian Xu, Linhao Liang

**Affiliations:** 1 School of Construction Machinery, Chang’an University, Xi’an 710064, China; 2017125042@chd.edu.cn (H.S.); 2017125005@chd.edu.cn (L.L.); 2 School of Management, Xi’an University Science and Technology, Xi’an 710054, China; jxu@xust.edu.cn

**Keywords:** subgrade, CDW, settlement prediction, the three-point hyperbolic combination model

## Abstract

Using construction and demolition waste (CDW) as road subgrade filling materials is an excellent way to solve the disparity between increased demand and road construction aggregate shortages. However, a key quality control problem is predicting the subgrade settlement, primarily because the CDW subgrade settlement prediction methods are not yet mature. To go some way in overcoming this problem, in this paper we developed a three-point hyperbolic combination model to predict CDW subgrade settlement, in which three appropriate points for the measured settlement curve were selected in the prediction samples to improve the hyperbolic model. Then, common prediction models—namely, the hyperbolic model, the three-point model, and the Hushino model—were compared with the proposed combination model to assess its viability. Finally, the three-point hyperbolic combination prediction accuracy was analyzed for different start points *t*_0_ and time intervals Δ*t*. The analyses found that the proposed model was in good agreement with the measured data, had a high correlation coefficient, and had only small errors. However, the time interval Δt needed to be greater than 80 days and the start point *t*_0_ needed to be selected at the beginning of the stable post-filling period, that is, *t*_0_ = 90–100 days. The application parameters were also determined to provide a reference for the large-scale application and settlement predictions of CDW subgrade.

## 1. Introduction

As a result of continued industrial and urban growth, there has been a commensurate increase in construction and demolition waste (CDW), which now accounts for 30–40% of city waste in China and more than 40% of all municipal waste in Europe [1,2,3]. Therefore, interest in recycling and reusing CDW has increased, and there have been significant achievements in CDW subgrade application. For example, it was found that CDW could be used as the raw material for the production of recycled aggregate concrete (RAC) for road embankments, subgrades, and foundations [4,5,6,7,8]. Li et al. [9] found that after a relatively simple treatment, CDW had high strength and was suitable for urban road embankments; it had an unconfined compressive strength under optimal moisture content from 0.85 to 0.62 MPa, with a mean value of 0.74 MPa, an average CBR value of at least 34.7%, an average embankment deflection of 0.66 mm, and a resilient modulus of 162.7 MPa—all of which met the relevant specifications.

The Xi’an–Xianyang North Ring Expressway is a key connection line for the “2367” expressway network in Shaanxi Province. To protect the environment and to reduce the construction waste in Xi’an and Xianyang, about 4 million tonnes of recycled CDW, or around 28% of the whole road subgrade project, is to be used in the construction of the expressway, most which is of collapsible loess foundation. As settlement and deformation are key structural factors affecting road structures, models are needed to calculate and predict CDW subgrade settlement and deformation. However, most current subgrade settlement research has been focused on soft soil subgrade, with little research having examined the settlement characteristics of CDW-filled subgrade or collapsible loess foundations. Therefore, the permanent deformation characteristics of CDW-filled subgrade need more detailed study, as large deformations can significantly decrease road life.

Soft soil subgrade settlement prediction algorithms can be divided into layerwise summation methods, numerical simulation methods, and curve fitting methods. Based on the foundation settlement depth range, layerwise summation methods divide the subgrade sections into several layers, and then calculate the compression in each layer to determine the total compression [10,11,12,13,14,15]. However, some of the methodological assumptions included in these methods are inconsistent with engineering practice and classical elastic solution assumptions. For example, as layerwise summation methods assume that the soil under the foundation has only vertical compressive deformation and no lateral deformation, the calculated settlements are often undervalued, and because it is also assumed that the foundations are flexible and have flexible loading, the foundation stiffness is often very large, which has obvious regulating effects on the settlement. Therefore, these types of assumptions mean that, to date, many lateral deformation settlement predictions have relatively large errors. 

Numerical simulations [16,17,18,19,20] are based on consolidation theory; that is, the calculations combine a constitutive material model, the final settlement, and development law. The parameter calculations are obtained based on indoor uniaxial consolidation and triaxial consolidation tests. Therefore, as these simulations are difficult to calculate, there are large calculation errors because of the boundary simplifications.

In order to avoid the disadvantages associated with the first two methods, the idea of a curve fitting method [21,22,23] was proposed, which involves predicting the permanent subgrade settlement based on measured settlement data in the early stages. Curve fitting methods—i.e., the Poisson model [24,25], the hyperbolic model [26,27], the three-point model [28,29], the Asaoka model [30,31], the Hushino model [32,33], the Gompertz model [34], the grey prediction model [35,36,37], and the neural network model [38,39]—are simple and easy to calculate, and have more satisfactory predictions as they fully consider the measured settlement data. However, as these models have some application limitations, accurate settlement prediction results for varied scenarios often require model combinations. For example, Yang et al. [40] employed a hyperbolic model, a settlement rate model, and a settlement difference model to predict soft soil embankment settlement, and found that the improved settlement difference model had the best fitting effect. Fan [41] used a logistic model and a Gompertz model to propose a combination forecasting model for the settlement prediction of soft soil foundations, and Li et al. [42] employed a combined prediction model based on an improved set pair analysis to predict subgrade settlement. Nejad et al. [43] proposed a back propagation neural network model to test the feasibility of predicting pile foundation settlements using artificial neural networks. Oliveira et al. [44] proposed a new improved quasi-equal time gray model (QGM (1,1)) prediction model to overcome the shortcomings of traditional non-isochronous models, such as cumbersome calculations and low medium- and short-term prediction accuracy. Zheng et al. [45] employed a Bayesian approach using laboratory data, field test data, and monitoring data, and achieved accurate predictions during the test embankment construction and consolidation periods in Ballina, New South Wales, Australia. Zhang [46] carried out four-factor, three-level orthogonal dynamic triaxial tests, calculated the Gray correlation degree and the influence degree for each permanent deformation factor, and then determined two different permanent deformation responses for CDW-filled subgrade, as well as the influence of the various factors. However, the suitability of these models for CDW subgrade settlement prediction needs further study, as the models must be systematically analyzed and optimized based on structural, geological, and settlement characteristics. 

Therefore, to meet the CDW subgrade settlement prediction requirements for the Xi’an–Xianyang North Ring Expressway, monitoring settlement data from the AK0+980 and AK1+130 CDW subgrade sections were used as the input data for the prediction model. Using the CDW subgrade combination forecasting settlement summing law, a three-point hyperbolic combination model was developed, in which the hyperbolic model was respectively optimized with the three-point model point selection. Four CDW subgrade settlement prediction methods were then compared: The proposed three-point hyperbolic combination model, the three-point model, the hyperbolic model, and the Hushino model; additionally, the practicability, limitations, and applicability of the proposed model were analyzed. Finally, to select the point characteristic parameters for the practical application of the three-point hyperbolic combination model, the start point *t*_0_ and the time span Δ*t* were studied to provide a theoretical basis for practical engineering embankment settlement and deformation predictions.

## 2. Three-Point Hyperbolic Combination Model for CDW Subgrade Settlement Prediction

Pan et al. [47] examined the theoretical bases, applicable conditions, and advantages and disadvantages of the three-point, hyperbolic, Poisson curve, and Asaoka models for foundation settlement predictions. It was found that as the three-point model accorded with Terzaghi’s one-dimensional consolidation theory, the physical quantity meant that the consolidation parameters could be easily obtained and parameter β had a clear physical meaning [48]. Therefore, the three-point model was found to have simple calculation advantages and a good adaptability to different curve shapes. While the hyperbolic model’s use of the graphic method to solve the parameters was found to be suitable for large deformation consolidation and settlement analysis, it was difficult to determine the consolidation parameters for the foundation. Zhao et al. [49] demonstrated that the actual settlement observation data–time curve for CDW subgrade conformed to the hyperbolic curve, with long-term observations from 1 to 5 years finding that the prediction settlement errors were only 0–2% in the later period. 

Therefore, based on the foundation characteristics of CDW subgrade, for the first time, this paper combined the characteristics of the three-point method and the hyperbolic model to propose a three-point hyperbolic combination model for CDW subgrade settlement predictions, for which relevant equations were designed and the prediction effects analyzed using case studies. 

### 2.1. Three-Point Model

The three-point model is also known as the logarithmic curve model of consolidation degree, the basic equation [28,29] for which is:

(1) $$S_{t}=S_{d}\alpha e^{-\beta t}+S_{\infty }(1-\alpha e^{-\beta t})$$

where *t* is the consolidation time, *S_t_* is the settlement value at *t*, *S_d_* is the instantaneous settlement value, and *S_∞_* is the final settlement value. Here, *α* takes an approximate value for the one-dimensional consolidation theory, that is, *α = 8/π^2^*, and *β* is the coefficient obtained from the regression of the measured data.

Three points (*S*_0_,*t*_0_), (*S*_1_*,t*_1_), (*S*_2_*,t*_2_) are chosen from the measured settlement–time curve (obtained from the measured data), with Δ*t* = *t*_2_ − *t*_1_ = *t*_1_ − *t*_0_. The values for the three points are then brought into Equation (1), and the simultaneous equations in Equations (2) and (3) are obtained:

(2) $$\beta =\frac{1}{t_{1}-t_{0}}ln\frac{s_{1}-s_{0}}{s_{2}-s_{1}},$$

(3) $$S_{d}=\frac{s_{0}-s_{\infty }(1-\alpha e^{-\beta t_{0}})}{\alpha e^{-\beta t_{0}}}.$$

### 2.2. Hyperbolic Model

The hyperbolic model assumes that the foundation settlement development law after the full load accords with the hyperbolic function, the equation for which is shown in Equation (4) [26]:

(4) $$S_{t}=S_{0}+\frac{t-t_{0}}{\alpha^{\prime }+\beta^{\prime }(t-t_{0})}$$

where *t*_0_ is the start time and $S_{0}$ is the settlement value at *t*_0_, with the start point being set after the full load. Therefore, $S_{t}$ is the settlement value at time *t*, and *α’* and *β’* are the parameters that need to be determined. 

Equation (4) is thus rewritten as Equation (5):

(5) $$\frac{t-t_{0}}{S_{t}-S_{0}}=\alpha^{\prime }+\beta^{\prime }(t+t_{0})$$

from which it can be seen that *α’* and *β’* are the intercept and the slope. The diagram for Equation (5) was determined using the linear fitting function in MATLAB R2016a software. By substituting the values for *α’* and *β’* into Equation (4), *S_t_* and *S_∞_* can be calculated.

### 2.3. Three-Point Hyperbolic Combination Model

Using the three-point method, $\left(t_{0},S_{0}\right)$, $\left(t_{1},S_{1}\right)$, $\left(t_{2},S_{2}\right)$ were taken from the measured data and Δ*t* = *t*_2_ − *t*_1_ = *t*_1_ − *t*_0_. Therefore, with *t*_0_ as the start point, the selected points *t*_1_ and *t*_2_ can be calculated using Equation (5), from which Equations (6) and (7) are obtained:

(6) $$\frac{t_{1}-t_{0}}{S_{1}-S_{0}}=\alpha^{\prime }+\beta^{\prime }(t_{1}+t_{0}),$$

(7) $$\frac{t_{2}-t_{0}}{S_{2}-S_{0}}=\alpha^{\prime }+\beta^{\prime }(t_{2}+t_{0}).$$

Equations (8) and (9) are then obtained by solving the simultaneous Equations (6) and (7):

(8) $$\alpha^{\prime }=\frac{2\Delta t(S_{2}-S_{1})}{\left(S_{2}-S_{0})(S_{1}-S_{0}\right)},$$

(9) $$\beta^{\prime }=\frac{2S_{1}-S_{2}-S_{0}}{\left(S_{2}-S_{0})(S_{1}-S_{0}\right)}.$$

Substituting the calculated values for *α’* and *β’* into Equation (4), the prediction settlement value at *t* is obtained, as shown in Equation (10):

(10) $$S_{t}=S_{0}+\frac{\left(t-t_{0})(S_{2}-S_{0})(S_{1}-S_{0}\right)}{2\Delta t(S_{2}-S_{1})+(2S_{1}-S_{2}-S_{0})(t-t_{0})}.$$

## 3. Case Study for the Three-Point Hyperbolic Combination Model

### 3.1. Actual Measured Settlement Data

The CDW subgrade settlement and deformation characteristics for the Xi’an–Xianyang North Ring Expressway were studied and predicted. The subgrade CDW, which came from a building waste recycling material factory in Xianyang, was fully utilized as embankment filler with a fill height of 5–6 m for the Xi’an–Xianyang North Ring Expressway. The main CDW aggregate composition was 63% concrete, 35% brick, 1% ceramic tile, and 1% other materials. The CDW aggregate particle size was 0–30 mm and the ratio of fine (passing through a 4.75 mm sieve) to coarse (retained by the 4.75 mm sieve) material was approximately 0.6 before the compaction. The subgrade filling comprised a 4:6 ratio of CDW and soil mixture that had a maximum dry density of 1.94 kN/m^3^ and an average moisture content of 12.4%, with the unconfined compression strength (UCS) and the California bearing ratio (CBR) under the average moisture content being 1.87 Mpa and 63.7%.

All of the CDW material properties fully met the subgrade filler requirements of the subgrade construction technical specifications. After tamping, the ultimate bearing capacity of the CDW subgrade foundations was 315.9 kPa under a consolidation degree condition of 55% and a consolidation pressure of 150 kPa; therefore, the subgrade foundation was rigid. In this study, two continuous sections (AK0+980 and AK1+130) in the experimental section were selected to measure the completed subgrade settlement, which had a subgrade fill height of 552.4 cm and a construction period from 21 March to 20 June 2016. The cumulative settlement for the actual settlement observations was measured at 23.9 mm and 23.8 mm after 360 days, the specific observations for which are shown in Table 1.

### 3.2. Settlement Characteristics Analysis

**Figure 1:**
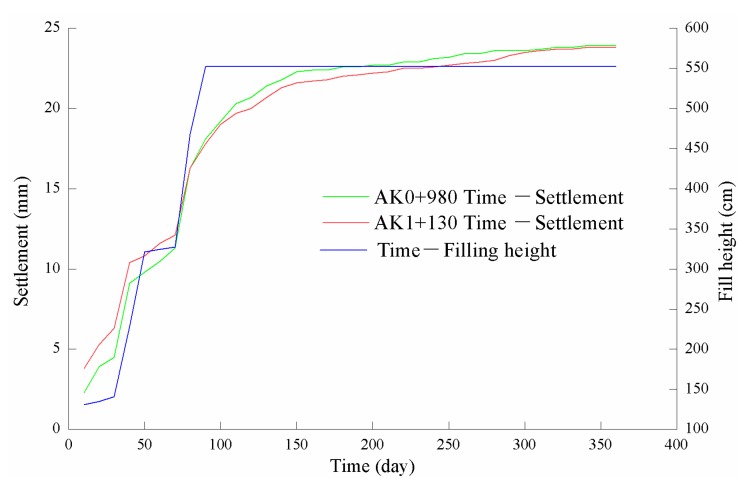
Construction and demolition waste (CDW) subgrade settlement trends.

From Table 1, the settlement data trend diagrams for AK0+980 and AK1+130 were drawn (Figure 1). The settlement curves increased rapidly in the early stages, slowed after a certain time, and finally tended toward the limit value; that is, the settlement went through four main process changes: occurrence, development, stability, and limit. Therefore, the soft soil embankment “S” growth model was found to also adequately describe the CDW subgrade settlement.

From the settlement observation data in Table 1, the CDW subgrade settlement characteristics were identified as having a small settlement deformation order of magnitude and a large relative fluctuation. For example, while the total settlement value for AK0+980 was only 23.9 mm, the maximum settlement value variations in the two adjacent observations were 5 mm; for example, the settlement value was 11.3 mm on the 70th day and 16.3 mm on the 80th day—a difference of 5 mm or 30.67% of the 16.3 mm cumulative observation. Therefore, it was concluded that the CDW subgrade settlement prediction algorithm should accurately predict the settlement trends by avoiding the influences caused by the fluctuations.

### 3.3. Settlement Prediction Results 

For AK0+980, *t*_0_, *t*_1_*,* and *t*_2_ were set at 90, 170, and 250 days, respectively, with the respective *S*_0_, *S*_1_*,* and *S*_2_ being 18.1, 22.4, and 23.2 mm. Using Equations (8) and (9) and with *α’* = 5.83675 and *β’ =* 0.159599, *α’* and *β’* were substituted into Equation (10) and the three-point hyperbolic model for AK0+980 determined as:

(11) $$S_{t}=18.1+\frac{t-90}{5.83675+0.159599(t-90)}.$$

After calculating *t* = 360 days, the predicted settlement for $S_{360d}$ for AK0+980 was 23.6183 mm, which‚ compared to the observation data (23.9 mm), indicated a prediction error of −1.18%.

Similarly, the *t*_0_, *t*_1_*,* and *t*_2_ for AK1+130 were set at 90, 170, and 250 days, respectively, with *S*_0_, *S*_1_*,* and *S*_2_ being 17.8, 21.8, and 22.7 mm. Using Equations (8) and (9), it was found that *α’* = 5.83675 and *β’ =* 0.159599. Then, substituting *α’* and *β’* into Equation (10), the three-point hyperbolic model for AK1+130 was:

(12) $$S_{t}=17.8+\frac{t-90}{9.81438+0.13441(t-90)}.$$

After calculating *t* = 360 days, the predicted settlement for $S_{360d}$ was 23.7033 mm, which, compared to the observation data (23.8 mm), indicated a prediction error of 0.41%.

**Figure 2:**
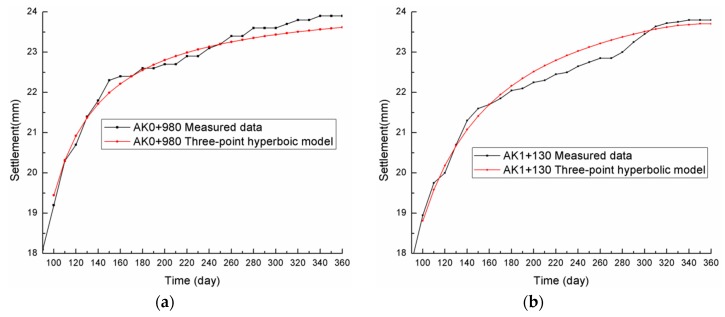
Comparison of the three-point hyperbolic model and the measured data: (**a**) AK0+980; (**b**) AK1+130.

Figure 2 shows the comparisons between the settlement prediction fitting curve and the actual observation settlement curve, from which it can be seen that the prediction curve was consistent with the measured data curve, and the three-point hyperbolic combination model had high fitting accuracy.

## 4. Discussion

### 4.1. Evaluation Indexes 

References [50,51] evaluated the applicability of several conventional settlement prediction models in terms of their correlation coefficients (*R*), sum of square prediction errors (*I_SSE_*), and relative deviations (*δ*), with the *R* being calculated using a correlation function, the *I_SSE_* being calculated as shown in Equation (13), and *δ* being calculated as shown in Equation (14).

(13) $$I_{SSE}=\sum_{i=1}^{n}S_{prediction}(i)-S_{Real}{\left(i\right)}^{2}$$

(14) $$\delta (i)=\left|S_{Prediction}(i)-S_{Real}(i)\right|/S_{Real}(i)$$

### 4.2. Comparison with Other Conventional Models 

To verify the validity and feasibility of the proposed combination model, the actual observation data in Table 1 were used as the prediction samples. As the Hushino model [52] has been widely applied to fit foundation settlement–time curves, the Hushino model, the three-point model, and the hyperbolic model were chosen for comparison with the developed three-point hyperbolic combination model.

The Hushino model is based on Terzaghi’s consolidation principle, which states that the consolidation degree (*U*) is the consolidation degree achieved after the external load is applied to the foundation. Therefore, as the consolidation degree is used to determine the relationships between the settlement and time, it is an important parameter for settlement prediction. The consolidation degree at *t* time (*U_t_*) is calculated using Equation (15) [52]:

(15) $$U_{t}=\frac{S_{t}}{S_{\infty }}$$

where *S_t_* is the settlement value at time *t* for the foundation soil, and *S_∞_* is the final foundation settlement value.

The Terzaghi consolidation principle states that when *U* is less than 60%, it is proportional to the square root of time. By studying the measured settlement values, it was concluded that the total settlement, including the shear deformation settlement, was proportional to the square root of time [53]. The Hushino formula is shown in Equation (16) [51]:

(16) $$S_{t}=S_{0}+S=S_{0}+\frac{AK\sqrt{t-t_{0}}}{\sqrt{1+K^{2}(t-t_{0})}}$$

where $S_{0}$ is the instantaneous settlement value, $S_{t}$ is the settlement value at time *t*, and *A* and *K* are the undetermined coefficients.

**Figure 3:**
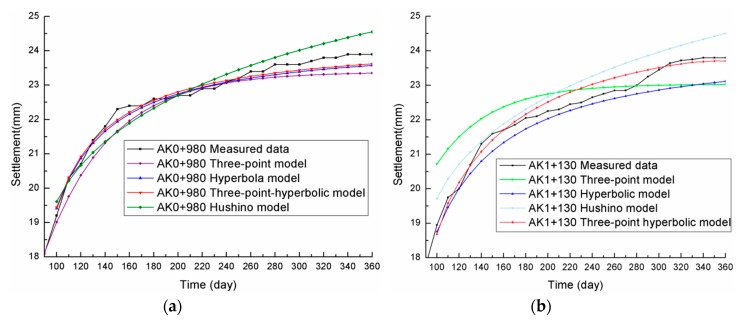
Comparison of the four prediction models: (**a**) AK0+980; (**b**) AK1+130.

Figure 3 compares the fitting data for the four settlement prediction models and the measured data, and Table 2 compares the settlement prediction evaluation indexes for the four models.

From Figure 3 and Table 2, the following could be concluded:

(1) The *I_SSE_* values for the Hushino model and the three-point model were larger than for the hyperbolic model and the three-point hyperbolic combination model, and the final settlement values also deviated significantly from the actual measured settlement curve. Therefore, as the Hushino model and the three-point model were found to have low CDW subgrade settlement predictive accuracy, they were deemed unsuitable, which was consistent with the results in Pan [51].

(2) The *I_SSE_* for the hyperbolic model was small and the *R* was high, which indicated that the hyperbolic model was also suitable for CDW subgrade settlement predictions.

(3) The predicted settlement value for the three-point hyperbolic combination model was the closest to the actual measured value, the *I_SSE_* was the smallest, and the *R* was the largest. Therefore, the three-point hyperbolic combination model was shown to be the best model for CDW subgrade settlement predictions as it had the highest accuracy. Furthermore, because the three-point hyperbolic combination model only used three of the measured values, data fluctuation was avoided, thereby making this model more feasible for CDW subgrade settlement predictions.

(4) As the three-point hyperbolic combination model is based on the selected three points, the calculations are simpler. However, as the CDW subgrade settlement observations had relatively large fluctuations, the selection of these three points is very important to ensure the best parameters and the most precise predictions. Therefore, to obtain optimum CDW subgrade prediction values and to avoid the errors caused by poor point selection, in the following, the three-point hyperbolic combination model prediction accuracy was examined using different time start points *t*_0_ and time intervals ∆*t*.

### 4.3. Influence of Time Intervals (Δt)

**Figure 4:**
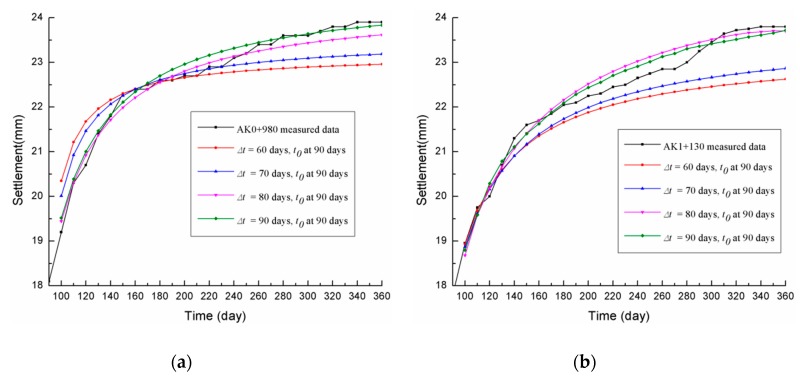
Comparison curves for the different time intervals (set *t*_0_ = 90 days): (**a**) AK0+980; (**b**) AK1+130.

The predicted three-point hyperbolic combination model results when the *t*_0_ was the same (*t*_0_ = 90 days, which was the subgrade filling completion date of June 21, 2016) and ∆*t* was set at 60, 70, 80, and 90 days are shown in Table 3, with the comparison between the prediction fitting curves and the measured data for the two sections (AK0+980 and AK1+130) shown in Figure 4.

From Table 3 and Figure 4, the following observations could be made:

(1) When the start point was *t*_0_ = 90 days, the smaller the ∆*t*, the larger the $I_{SSE}$, the higher the δ_360*d*_, and the lower the *R*. In contrast, the greater the ∆*t*, the closer the three-point hyperbolic model fitting curve was to the actual measured curve, the higher the *R*, the smaller the $I_{SSE},$ and the lower the δ_360*d*_.

(2) For AK0+980, when *t*_0_ was set at 90 days and ∆*t* was set at 60, 70, 80, and 90 days, the correlation coefficients were 0.97183, 0.98195, 0.99197, and 0.99205, respectively, and the respective *I_SSE_* values were 10.90904, 5.95313, 1.4755, and 0.85920. Therefore, to improve the prediction accuracy, ∆*t* should be no less than 80 days

(3) Similarly, when *t*_0_ was set at 90 days and ∆*t* was set at 60, 70, 80, and 90 days, the correlation coefficients of AK1+130 were 0.97995, 0.98336, 0.98713, and 0.98793, respectively, and the respective *I_SSE_* values were 13.06036, 8.14553, 3.32137, and 2.817681. Therefore, the best ∆*t* should be no less than 80 days, which is consistent with the parameter for AK0+980.

### 4.4. Influence of Different Start Points (t_*0*_)

**Figure 5:**
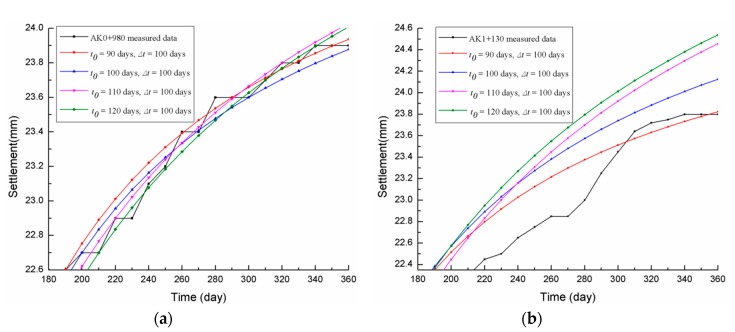
Comparison curves for different start points (set Δt = 100 days): (**a**) AK0+980; (**b**) AK1+130.

Table 4 shows the three-point hyperbolic combination model fitting calculations for when the time interval Δt was the same (set to Δt = 100 days) and *t*_0_ was set at 90 days (completion date), 100, 110, and 120 days. The comparisons between the settlement prediction curves for the two sections (AK0+980 and AK1+130) and the measured data curves are shown in Figure 5.

From Table 4 and Figure 5, the following could therefore be concluded:

(1) At Δt = 100 days, the larger the *t*_0_, the larger the δ_360*d*_ and $I_{SSE}$, and the lower the *R*. On the contrary, the smaller the *t*_0_, the closer the three-point hyperbolic model fitting curve was to the measured curve, the higher the *R*, and the smaller the δ_360*d*_ and $I_{SSE}$.

(2) For AK0+980, when Δt = 100 days and *t*_0_ was set at 90, 100, 110, and 120 days, the correlation coefficients were 0.99049, 0.98889, 0.98614, and 0.98217, respectively, and the respective *I_SSE_* values were 1.18017, 1.29816, 1.31485, and 1.67211. Therefore, it is recommended that the start point *t*_0_ be at the beginning of the stable period after the subgrade has finished.

(3) Similarly, when Δt = 100 days and *t*_0_ was set at 90, 100, 110, and 120 days, the correlation coefficients of AK1+130 were 0.98825, 0.98688, 0.98383, and 0.98361, respectively, and the respective *I_SSE_* values were 1.57992, 3.21601, 6.69725, and 7.201311. Therefore, the best *t*_0_ should be at the beginning of the stable period after the subgrade has finished, which is consistent with the parameter for AK0+980.

## 5. Conclusion

(1) The CDW subgrade consolidation settlement was found to be in accordance with the soft soil embankment “S” growth model, except that it had a smaller settlement deformation order of magnitude and a larger relative fluctuation.

(2) A three-point hyperbolic combination model was proposed to predict CDW subgrade settlement. Compared with other traditional models—namely, the three-point model, the hyperbolic model, and the Hushino model—the three-point hyperbolic combination model had the highest *R* at 0.99197, the smallest *I_SSE_* at 1.477547 mm^2^, and the closest predicted settlement value $S_{360d}$, at 23.61826 mm, to the actual measured value (23.90 mm). As the comparison curves showed that the prediction curve for the three-point hyperbolic combination model was in good agreement with the measured data, it was concluded that the proposed combination model had the best adaptability for CDW subgrade settlement prediction.

(3) To improve the prediction accuracy of the proposed combination model, the effects of different time intervals Δt and start points *t*_0_ on the prediction accuracy were analyzed, and it was found that Δt should be set at no less than 80 days and *t*_0_ needs to be selected at the beginning of the stable period after the subgrade fill completion (that is, *t*_0_ = 90 or 100 days).

(4) The three-point hyperbolic combination model provides a theoretical basis for CDW subgrade settlement and deformation predictions, and especially for collapsible loess foundations, as it is able to provide a scientific basis for the application of CDW subgrade filling materials. The application of the combination model, therefore, can promote large-scale CDW applications and can support sustainable development aims.

## Figures and Tables

**Table 1:** Actual settlement measured data of the two sections.

Time (Day)	AK0+980 Settlement (mm)	AK1+130 Settlement (mm)	Fill Height (cm)	Time (Day)	AK0+980 Settlement (mm)	AK1+130 Settlement (mm)	Fill Height (cm)
10	2.3	3.8	130.9	190	22.6	22.1	552.4
20	3.9	5.3	134.9	200	22.7	22.2	552.4
30	4.5	6.3	140.8	210	22.7	22.3	552.4
40	9.1	10.4	228.4	220	22.9	22.5	552.4
50	9.8	10.8	321.4	230	22.9	22.5	552.4
60	10.5	11.6	324.7	240	23.1	22.6	552.4
70	11.3	12.1	327.4	250	23.2	22.7	552.4
80	16.3	16.3	468.1	260	23.4	22.8	552.4
90	18.1	17.8	552.4	270	23.4	22.9	552.4
100	19.2	19	552.4	280	23.6	23.0	552.4
110	20.3	19.7	552.4	290	23.6	23.3	552.4
120	20.7	20	552.4	300	23.6	23.5	552.4
130	21.4	20.7	552.4	310	23.7	23.6	552.4
140	21.8	21.3	552.4	320	23.8	23.7	552.4
150	22.3	21.6	552.4	330	23.8	23.7	552.4
160	22.4	21.7	552.4	340	23.9	23.8	552.4
170	22.4	21.8	552.4	350	23.9	23.8	552.4
180	22.6	22.0	552.4	360	23.9	23.8	552.4

**Table 2:** Evaluation indexes’ comparison of the four prediction models.

Section	Category	Three-Point Model	Hyperbolic Model	Hushino Model	Three-Point Hyperbolic Combination Model
AK0+980	Initial parameters	*t*_0_ = 90 days Δ*t* = 80 days	*t*_0_ = 90 days *S*_0_ = 18.1 mm	*t*_0_ = 90 days *S*_0_ = 18.1 mm	*t*_0_ = 90 days Δ*t* = 80 days
	*S_360d_* (mm)	23.3483	23.5760	24.5750	23.6183
	*R*	0.98753	0.99175	0.97443	0.99197
	*I_SSE_* (mm^2^)	3.65791	1.24474	3.58260	1.47755
	$\delta_{360d}(\%)$	2.31	1.36	2.82	1.18
AK1+130	Initial parameters	*t*_0_ = 90 days Δ*t* = 80 days	*t*_0_ = 90 days *S*_0_ = 17.8 mm	*t*_0_ = 90 days *S*_0_ = 17.8 mm	*t*_0_ = 90 days Δ*t* = 80 days
	*S_360d_* (mm)	23.0168	23.1168	24.5033	23.7033
	*R*	0.97458	0.98723	0.98861	0.98713
	$I_{SSE}\left({\mathrm{mm}}^{2}\right)$	4.32954	5.16570	7.83759	3.32137
	$\delta_{360d}(\%)$	3.29	2.87	2.96	0.41

**Table 3:** Comparison of the prediction accuracy for different time intervals (*t_0_* = 90 days).

Section	Δt (day)	*R*	$I_{SSE}$ $\left(mm^{2}\right)$	$S_{360d}\;(\mathrm{mm})$	$\delta_{360d}\;\left(\%\right)$
AK0+980	60	0.97183	10.90904	22.9570	3.95
	70	0.98195	5.95313	23.1847	2.99
	80	0.99197	1.47755	23.6183	1.18
	90	0.99205	0.85920	23.9452	0.19
AK1+130	60	0.97995	13.06036	22.6245	4.94
	70	0.98336	8.14553	22.8656	3.93
	80	0.98713	3.32137	23.7033	0.41
	90	0.98793	2.81768	23.7157	0.35

**Table 4:** Comparison of the prediction accuracy for different start points (Δt = 100 days).

Section	$t_{0}\;(\mathrm{day})$	R	$I_{SSE}\;\left(mm^{2}\right)$	$S_{360d}\;(\mathrm{mm})$	$\delta_{360d}\;\left(\%\right)$
AK0+980	90	0.99049	1.18017	23.9362	0.15
	100	0.98889	1.29816	23.8776	0.09
	110	0.98614	1.31485	24.0268	0.53
	120	0.98217	1.67211	24.0091	0.46
AK1+130	90	0.98825	1.57992	23.8233	0.10
	100	0.98688	3.21601	24.1256	1.37
	110	0.98383	6.69725	24.4582	2.77
	120	0.98361	7.20131	24.5390	3.11
